# Pharmacist Prescriptive Authority: Lessons from Idaho

**DOI:** 10.3390/pharmacy8030112

**Published:** 2020-07-08

**Authors:** Alex J. Adams

**Affiliations:** Idaho State Board of Pharmacy, Boise, ID 83702, USA; alexadamsrph@gmail.com; Tel.: +1-419-708-5186

**Keywords:** advanced pharmacy practice, scope of practice, pharmacist prescriptive authority

## Abstract

Pharmacist prescriptive authority continues to increase at the state level in the United States. Recently, the Idaho Board of Pharmacy (BOP) finalized regulations that expanded autonomous prescriptive authority in its state to a range of preventative care as well as acute and chronic conditions. This manuscript reviews the key decision points made by the BOP regarding drug categories included, education requirements, protocols, access to data, and use of standards of care. Overall, Idaho’s approach closely reflects the medical model of regulation and may prove useful to other states and jurisdictions as they consider similar issues.

## 1. Introduction

Pharmacist prescriptive authority continues to increase at the state level in the United States. While the recent attention might suggest this is a new phenomenon, pharmacists have had the authority to prescribe in at least some states for four decades, traditionally under a collaborative practice agreement (CPA). In a CPA, a physician (or other practitioner) establishes parameters for pharmacists to initiate or modify medication regimens under certain conditions [1,2]. Nearly all states (49) and the District of Columbia currently allow pharmacists to prescribe under a CPA, and an increasing body of evidence has demonstrated that patient outcomes improve when pharmacists are fully practicing to the extent of their clinical abilities [3,4,5].

States have recently advanced autonomous models of pharmacist prescriptive authority that are not preconditioned on a pharmacist first finding a willing partner and entering into a CPA. Two primary models of autonomous pharmacist prescribing have been advanced: (1) statewide protocols; and (2) unrestricted category-specific authority [6]. In the former, a state agency (such as a board of pharmacy or department of health) publishes a protocol that any qualified pharmacist is permitted to follow. The protocol is non-negotiable at the practice level, and the state must continuously update it if practice guidelines change. In the latter model, pharmacists have true independent prescriptive authority, limited to certain classes of medications.

While CPAs have formed the historical basis for advanced pharmacist roles in ambulatory care and institutional practice settings, they have been less common in community pharmacy settings [2]. This is in part due to the difficulty in finding a willing collaborator and aligning incentives among providers who may view each other as competitors for certain services [7]. Autonomous models of pharmacist prescriptive authority have thus enabled patient access to services that *could* have been provided under a CPA, but were not widely available due to the practical limitations inherent in any model in which one professional’s authority is dependent on the permission of another potentially-competing professional.

Autonomous models of pharmacist prescribing have sparked some of the most significant public health achievements of the pharmacy profession in recent years, such as prescribing and administering immunizations. The Centers for Disease Control and Prevention (CDC) has lauded the profession’s efforts to increase vaccination rates in the United States [8]. One-third of all influenza vaccines given during a recent flu season were provided in a community pharmacy [9]. In addition, pharmacist prescribing of naloxone has significantly increased co-prescribing of this critical antidote in the midst of a nationwide opioid epidemic [10].

Recently, the Idaho Board of Pharmacy (BOP) finalized regulations that expanded autonomous prescriptive authority in its state to a range of preventative care as well as acute and chronic conditions [11]. The prescriptive authority was conditioned on pharmacists following the applicable standard of care that would be provided by another prudent provider in the same or similar setting. Idaho’s unique approach has generated much discussion, and this manuscript aims to summarize both the historical context for the new regulations, and several of the key decision points the BOP considered in finalizing its regulations. Our hope is that this manuscript will prove useful for other states considering similar issues.

## 2. Legislative and Regulatory History of Pharmacist Prescribing in Idaho

The American College of Clinical Pharmacy (ACCP) has put forth a definition of prescribing as a broad set of medication-related activities: selecting, initiating, monitoring, continuing, discontinuing, modifying, and/or administering drug therapy [12]. Using this definition, Idaho pharmacists have been able to prescribe since at least 1998 under a CPA [13]. Specifically, Idaho pharmacists had the authority to “initiate and modify drug therapy management” within a protocol established with a collaborating prescriber. Idaho originally had a patient-specific CPA law that required the prescribing practitioner to first refer the patient to a pharmacist; this was broadened in subsequent years to become a population-specific CPA law as the BOP and legislature became comfortable with the model [11].

The first foray into autonomous pharmacist prescribing in Idaho occurred in 2011 when the BOP brought forth House Bill 218. This bill amended the definition of the “practice of pharmacy” to include autonomous prescribing of immunizations for persons aged 12 and older, and dietary fluoride supplements [14]. Previously, a CPA was necessary for pharmacists to prescribe these medications, and this rate-limiting step was removed. The legislative testimony on HB 218 focused on the public health needs of the state. Namely, the immunization authority came on the heels of the 2009 H1N1 influenza pandemic, during which state and federal public health officials lauded the convenience and accessibility of pharmacists as an opportunity to “extend the reach” of public health [15]. Proponent testimony highlighted how this bill could help improve Idaho’s low vaccination rate [16]. Similarly, the inclusion of fluoride supplements was championed by a dentist because of the need in some rural communities [17,18].

In 2015, opioid antagonists were added to the list of pharmacist prescriptive authority in the statutory definition of pharmacy practice, and a year later, epinephrine auto-injectors were added [19,20,21]. In both cases, pharmacists could prescribe these products not just to a patient in need, but to any person or entity in a position to assist a patient. In proponent testimony, it was suggested that increased naloxone access could help prevent opioid overdose deaths, particularly in rural areas [22]. Similarly, the epinephrine bill was described as a way to increase access to care in venues in which patients may experience anaphylaxis [23]. Of note, both bills originated outside the profession of pharmacy: naloxone by a legislator (and supported by various public health entities), and epinephrine by a patient advocacy group focused on food allergies. Also in 2016, the immunization prescribing authority was modified to lower the patient age threshold, allowing pharmacists to now prescribe immunizations to individuals age six or older [24].

Early in the 2017 legislative session, bills passed adding tuberculin purified protein derivative products and all tobacco-cessation products (inclusive of bupropion and varenicline) to the statutory prescribing list [25,26,27,28]. Proponent testimony again focused on the public health benefit of increasing access to tobacco-cessation drugs [29]. During the legislative committee hearing on the tobacco-cessation bill, committee members inquired about other medications that pharmacists could prescribe, and commented on the piecemeal nature of bringing a separate bill for each individual medication class as a proposed addition [30]. 

Shortly thereafter, a bipartisan group of legislators co-sponsored House Bill 191, which sought to change the process by which determinations around pharmacist prescriptive authority are made moving forward [31]. Rather than the legislature continuing to make determinations on pharmacist prescribing in a piecemeal fashion, the bill granted the BOP rulemaking authority to add drugs, drug categories, or devices to the prescribing list as long as one of the following four conditions was met:

1. A new diagnosis is not required;
2. The condition to be treated is minor and generally self-limiting;
3. The condition has a CLIA-waived test to guide diagnosis; or
4. There is an emergency situation, whereby the patient’s health or safety is threatened without immediate access to a prescription.

House Bill 191 explicitly prohibited the board from adding controlled drugs, compounded drugs, or biological products to the independent prescribing list as part of the rulemaking process [31]. The bill was signed into law on 24 March 2017, and the BOP’s rulemaking was initiated soon thereafter.

The BOP finalized Rule Docket 27.01.04, Rules Governing Pharmacist Prescriptive Authority, which took effect on 1 July 2018 [11]. The rules added more than 20 drug and device categories to the list of pharmacist prescriptive authority (Table A1). To populate this initial drug list, the BOP drew heavily from examples in other states as well as minor ailment prescribing programs in Canada and elsewhere [32]. The BOP focused on drug classes that could improve public health, and for which pharmacists had a proven track record of prescribing safely and effectively in other jurisdictions. 

The BOP rules also created a general prescribing framework that applied to all drug categories (Table A2). This framework was subject to legislative review in January 2018 and again in 2019. Given the perceived strength of this framework, the Idaho legislature passed House Bill 182 in 2019, removing the requirement that the BOP must adopt specific rules for each drug or drug category that pharmacists may prescribe. As a result, pharmacists can prescribe any drug for prevention for conditions that do not require a new diagnosis, is used in an emergency, is for a minor/self-limiting condition, or can be diagnosed through a CLIA-waived test. 

## 3. Pharmacist Prescribing: Key Decision Points

The BOP’s statutory mission is to protect the health, safety, and welfare of the public through the effective regulation of the practice of pharmacy. As such, the BOP was faced with a series of decisions as it established general requirements (Table A2) for pharmacist prescribing under its new authority. The key decision points are discussed below.

### 3.1. Assimilate into Existing Prescribing Practices 

The BOP felt that pharmacists acting as prescribers should generally assimilate into the existing practices of other prescribers. For example, Idaho law sets parameters for self-prescribing and what elements must be on a valid prescription drug order; we chose to hold pharmacists accountable to these existing prescriber rules (and others) rather than attempting to create pharmacy-specific ones.

### 3.2. Drugs vs. Drug Categories 

The BOP followed the model established in Canada and generally listed drug categories by the condition they intend to treat as opposed to individual drugs (e.g., ‘drugs approved for cold sores’ vs. valacyclovir). Doing so prevented the BOP from having to update the rules every time guidelines change or new agents are approved by the FDA. Further, individual drugs have multiple uses, and listing drug categories by intended condition was thus seen as the preferable option.

### 3.3. Education Requirements 

The BOP felt strongly that prescribing should be limited to pharmacists who are educationally prepared and for whom competence has been both achieved and maintained. It stopped short, however, of setting advanced credentialing requirements as a matter of law. For one, states that have set such requirements such as residency completion and board certification for their CPA models have found that it significantly limits uptake [33]. More importantly, however, the BOP saw little connection between the training provided by an inpatient residency and the skills necessary for a community pharmacist to assess a patient for a cold sore, as one example. The published literature demonstrated that community pharmacists were able to successfully achieve patient care outcomes with skill-specific or refresher continuing education. Lastly, institutional credentialing and privileging is a risk-mitigation strategy within the pharmacy profession, as it is within other health professions, even in the absence of specific legal requirements [34].

### 3.4. Recognizing Symptoms Necessitating Referral 

While many would generally agree that pharmacists could safely treat some minor ailments, a common concern revolved around whether pharmacists could appropriately recognize symptoms that should suggest a referral to more advanced medical care. As one physician recently put it, how do you “know when a sore throat is a sore throat and when it’s really cancer [35]”.

Published studies demonstrate that protocols help pharmacists identify which patients may be safely treated in a pharmacy versus those who may need to be seen by another medical professional [36,37,38,39,40,41,42,43]. Pharmacists have a history of successfully using protocols to identify appropriate candidates for treatment while referring patients when appropriate because of the presence of certain high-risk factors. Given their basis in published literature and their similarity to the CPA approach, the BOP required prescribing pharmacists to use a patient assessment protocol, even though we were not aware of this being a requirement of any other health profession in Idaho.

### 3.5. Specific Protocol vs. Template Protocol 

There was considerable discussion over whether to mandate the use of a specific statewide protocol. Some states have mandated the use of specific protocols for immunizations and naloxone, though Idaho had not done so and still achieved its public health aims.

The BOP found that protocols were generally already available for most of the drug categories that it was considering [44,45]. In addition, by calcifying the protocols in state law, it would require the BOP to engage in rulemaking each time new studies were published, or if clinical guidelines were updated. Thus, rather than mandating the use of a specific statewide protocol, the BOP set its expectation that pharmacists use a protocol that is “based on current clinical guidelines, when available, or evidence-based research” as it relates to inclusion, exclusion, and referral criteria. As a compromise, the BOP worked with diverse stakeholders to issue *template* protocols as a starting point for pharmacists to fulfill their obligations under the general requirements, though pharmacists must “revise the patient assessment protocol when necessary to ensure continued compliance with clinical guidelines or evidence-based research findings [46]”.

### 3.6. Access to Data 

The BOP felt strongly that pharmacists should only prescribe when they had access to sufficient information to justify the care provided and established this expectation as part of the documentation requirements. The BOP’s template protocols establish a range of referral criteria based off of published literature. For example, based on the template protocols, a patient presenting with influenza-like illness should be referred if their oxygenation is less than 90% via pulse oximetry; if a pharmacy does not have a means of determining this, the pharmacist should not prescribe for influenza.

### 3.7. Coordination of Care 

The BOP was aware of the fragmentation of care that can occur when patients seek care at venues like urgent care facilities and retail clinics. To ensure that care provided at pharmacies is better coordinated with the broader medical team, the BOP required pharmacists to provide notification of care provided to the patient’s primary care provider (PCP), though we were not aware of other health professions or settings that had such a legal requirement [47]. If the patient does not have a PCP—which studies suggest will occur in approximately 25% of the patients who seek care for minor ailments at the pharmacy—the BOP encourages pharmacies to partner with the medical community and provide lists of PCPs who are enrolling new patients in the local community [36].

### 3.8. Conflict of Interest 

Idaho is a state that allows physicians to dispense outpatient drugs; in fact, the state has more licensed physician dispensing outlets than licensed retail pharmacies in the state. While the BOP had not received complaints about potential conflicts of interest that result from physicians simultaneously prescribing and dispensing, it sought to set a high bar and built in multiple accountability mechanisms for pharmacists who intend to do the same. Namely, the BOP requires real-time electronic recordkeeping systems which facilitate real-time claims adjudication; this leverages the claims edits of health plans such as early refill and duplicative therapy warnings. In addition, the BOP augmented its regulations regarding unprofessional conduct, allowing it to pursue disciplinary cases against pharmacists who are “promoting or inducing…health care services or products that are unnecessary or not medically necessary”.

### 3.9. Standard of Care 

Lastly, the BOP augmented its disciplinary authority against pharmacists who provide services which “fail to meet the standard provided by other qualified licensees…in the same or similar setting”. Thus, if a pharmacist prescribed a statin and failed to check requisite laboratory tests, the BOP could pursue discipline in such an instance for failing to uphold the applicable standard of care [48]. Thus, rather than specifying in law which tests are needed, or what referral thresholds must be followed, the BOP adopted a standard-of-care approach that has been successfully leveraged by the medical and nursing professions. By adopting a standard-of-care model, the law is flexible to change with new research and new guidelines, and the BOP will not have to continuously update its rules to keep pace with change.

## 4. Discussion

The journey to prescriptive authority in Idaho evolved over a 20 year period and provides potential lessons for other states. The cascade started with CPAs and then, after a successful track record with this approach, policy makers felt comfortable pulling specific drug classes out of this dependent authority and into an autonomous model. The piecemeal legislative approach aimed to address specific public health needs (e.g., low immunization rates, low opioid antagonist co-prescribing in an opioid epidemic, and low tobacco-cessation rates). After a successful track record with these autonomous drug classes, a bill empowered the BOP to promulgate rules within defined parameters to further populate the autonomous prescribing list. Eventually, this requirement for rulemaking was removed and Idaho pharmacists now have broad authority to prescribe in line with the general prescribing framework described in Table A2.

Several key themes may prove useful for other states. First, the identification and cultivation of supporters external to the profession of pharmacy was critical. Several of the piecemeal legislative bills originated outside the profession from both legislators and patient advocacy groups. Even those bills that were brought forth by the BOP received support from public health stakeholders or other health professions (e.g., a dentist championing fluoride prescribing). This support was undoubtedly an important success factor.

Second, leverage the experience and evidence of other jurisdictions. Pharmacists have been prescribing autonomously in Canada, the United Kingdom, and several states for a number of years. Learning from their experiences, particularly their published research, can ensure that the best available evidence is used to inform the debate rather than just speculation. Given the convenience and accessibility of pharmacists, it is easy for some to see expanded pharmacist roles as a tradeoff of increasing access at the expense of safety. The BOP considered increased access as a positive corollary to pharmacist prescribing, but reviewed literature for safety and effectiveness alone, and primarily considered the addition of drugs for which pharmacists had demonstrated success prescribing in other jurisdictions.

Lastly, adopting a standard-of-care approach may be a mechanism to ensure public safety while enabling flexibility in practice. In the context of medical regulation, this term generally refers to “that which a minimally competent physician in the same field would do under similar circumstances [49]”. A regulatory model based on the standard of care is more flexible and is determined by the individual circumstances that present in practice rather than specific requirements codified in law. In May 2018, the National Association of Boards of Pharmacy (NABP) established a task force to help states develop regulations based on standards of care rather than “prescriptive rule-based regulation” and thus we anticipate that other states will take this approach in the coming years [50,51].

Since taking effect, several national and regional pharmacy chains have issued press releases indicating that they are prescribing a subset of the medications or devices included in the Idaho prescribing rules [52,53,54,55,56]. To date, no patient safety concerns have been raised with the BOP, and numerous positive anecdotes have been relayed. For example, one pharmacist relayed a story about a tourist in an Idaho resort town who sought advice about her symptoms suggestive of an uncomplicated urinary tract infection. The town does not have an urgent care facility, and the patient was faced with the prospects of going to an out-of-state emergency department; the pharmacist instead leveraged an evidence-based protocol, assessed the patient, determined that the patient did not meet any referral criteria, and therefore provided the needed medication to the patient onsite, saving the patient both time and money.

Overall, Idaho’s approach closely reflects the medical and nursing model of regulation, buttressed by the general pharmacist prescribing requirements established in law. The BOP’s rules and approach may prove useful to other states and jurisdictions as they consider similar issues.

## Appendix A

**Table A1:** Drugs and Devices that Idaho Pharmacists May Prescribe by Rule ^a^.

Category	Drug, Drug Category, or Device
Non-Prescription Drugs and Devices (Rule 020)	Any non-prescription drug or device
Minor Conditions (Rule 021)	Any FDA-approved drug indicated for: Lice Cold sores Motion sickness prevention Uncomplicated urinary tract infections Allergic rhinitis Mild acne Mild cough (specifically benzonatate)
Devices (Rule 022)	Inhalation spacer Nebulizer Diabetes blood sugar-testing supplies Pen needles Syringes
CLIA-Waived Test (Rule 023)	Any FDA-approved drug indicated for the following conditions, provided the patient first tests positive on a CLIA-waived test: Influenza Group A streptococcal pharyngitis
Gaps in Care (Rule 024)	Statins, for patients who have been diagnosed with diabetes Short-acting beta agonists (SABA), for patients with asthma who had a prior prescription for a SABA, and who have a current prescription for a long-term asthma control medication
Travel Drugs (Rule 025)	Any non-controlled drug in the CDC Yellow Book
Supplement to an Infusion Order (Rule 026)	Any of the following FDA-approved drugs or devices may be added as a supplement to a valid infusion order: Heparin flush Infusion pumps and other rate control devices Tubing, filters, catheters, IV start kits, central line dressing kits, and injection caps Local anesthetics for IV port access Agents for catheter occlusion Additional supplemental drugs (specifically methylprednisolone, hydrocortisone, diphenhydramine, epinephrine, and normal saline)
Emergency Drugs (Rule 027)	In an emergency, after contacting emergency medical services, the following FDA-approved drugs: Diphenhydramine Epinephrine SABA
Lyme Disease Prophylaxis (Rule 028)	Antimicrobial prophylaxis

(^a^) In addition to those allowed in rule, Idaho pharmacists had statutory authority to prescribe immunizations, dietary fluoride supplements, opioid antagonists, epinephrine auto-injectors, tuberculin purified protein derivative, and tobacco-cessation drugs.

**Table A2:** General Requirements for Pharmacist Prescribing.

Core Element.	Original Regulatory Language
Education	The pharmacist may only prescribe drugs or devices for conditions for which the pharmacist is educationally prepared and for which competence has been achieved and maintained.
Patient–Prescriber Relationship	The pharmacist may only issue a prescription for a legitimate medical purpose arising from a patient–prescriber relationship as defined in Section 54-1733, Idaho Code.
Patient Assessment Protocol	The pharmacist must obtain adequate information about the patient’s health status to make appropriate decisions based on the applicable standard of care. At a minimum, for each drug or drug category the pharmacist intends to prescribe, the pharmacist must maintain a patient assessment protocol based on current clinical guidelines, when available, or evidence-based research findings that specifies the following: Patient inclusion criteria, and Explicit exclusion and medical referral criteria. The pharmacist must revise the patient assessment protocol when necessary to ensure continued compliance with clinical guidelines or evidence-based research findings. The pharmacist’s patient assessment protocol, and any related forms, must be made available to the Board upon request.
Collaboration with Other Health Care Professionals	The pharmacist must recognize the limits of the pharmacist’s own knowledge and experience and consult with and refer to other health care professionals as appropriate.
Follow-Up Care Plan	The pharmacist must develop and implement an appropriate follow-up care plan, including any monitoring parameters, in accordance with clinical guidelines.
Notification	The pharmacist must inquire about the identity of the patient’s primary care provider; and, if one is identified by the patient, provide notification within five (5) business days following the prescribing of a drug. In the instance in which the pharmacist is prescribing to close a gap in care or to supplement a valid prescription drug order, the pharmacist must alternatively notify the provider of record.
Documentation	The pharmacist must maintain documentation adequate to justify the care provided, including, but not limited to, the information collected as part of the patient assessment, the prescription record, any notification provided as required under this section, and the follow-up care plan.

## References

[1] National Alliance of State Pharmacy Associations (NASPA) (2015). Pharmacist Collaborative Practice Agreements: Key Elements for Legislative and Regulatory Authority. https://naspa.us/wp-content/uploads/2017/01/CPA-Workgroup-Report-FINAL.pdf.

[2] Bacci J.L., Coley K.C., McGrath K., Abraham O., Adams A.J., McGivney M.S. (2016). Strategies to facilitate the implementation of collaborative practice agreements in chain community pharmacies. J. Am. Pharm. Assoc..

[3] Giberson S., Yoder S., Lee M.P. (2011). Improving Patient and Health System Outcomes through Advanced Pharmacy Practice. A Report to the U.S. Surgeon General.

[4] Centers for Disease Control and Prevention (CDC) Advancing Team-Based Care Through Collaborative Practice Agreements: A Resource and Implementation Guide for Adding Pharmacists to the Care Team. https://www.cdc.gov/dhdsp/pubs/docs/CPA-Team-Based-Care.pdf.

[5] National Governors Association (NGA) (2015). The Expanding Role of Pharmacists in a Transformed Health Care System. NGA Paper. https://classic.nga.org/files/live/sites/NGA/files/pdf/2015/1501TheExpandingRoleOfPharmacists.pdf.

[6] Adams A.J., Weaver K.K. (2016). The Continuum of Pharmacist Prescriptive Authority. Ann. Pharmacother..

[7] Adams A.J., Dering-Anderson A., Klepser M.E., Klepser D. (2018). The Roles of Pharmacy Schools in Bridging the Gap Between Law and Practice. Am. J. Pharm. Educ..

[8] Schuchat A. (2015). Letter to Pharmacists. https://www.pharmacist.com/sites/default/files/files/CDC%20letter%20to%20pharmacists%20vaccinators.pdf.

[9] Lutz R. (2015). Pharmacist-Provided Flu Shots Please Patients. Pharmacy Times. http://www.pharmacytimes.com/resource-centers/cough-cold/pharmacist-provided-flu-shots-please-patients.

[10] Gertner A.K., Domino M.E., Davis C.S. (2018). Do naloxone access laws increase outpatient naloxone prescriptions? Evidence from Medicaid. Drug Alcohol Depend..

[11] Idaho State Board of Pharmacy Rule Docket 27-0104-1701. Rules Governing Pharmacist Prescriptive Authority. https://adminrules.idaho.gov/rules/current/27/270104.pdf.

[12] Carmichael J.M., O’Connell M.B., Devine B., Kelly H.W., Ereshefsky L., Linn W.D., Stimmel G.L. (1997). Collaborative drug therapy management by pharmacists. American College of Clinical Pharmacy. Pharmacother. J. Hum. Pharmacol. Drug Ther..

[13] 1998 Idaho Administrative Code Rules of the Board of Pharmacy. Rule 165. Pharmacotherapy. https://adminrules.idaho.gov/rules/1999/27/0101.pdf.

[14] Idaho State Legislature House Bill 218. 2011 Legislative Session. https://legislature.idaho.gov/sessioninfo/2011/legislation/H0218/.

[15] Rosenfeld L.A., Etkind P., Grasso A., Adams A.J., Rothholz M.C. (2011). Extending the reach: Local health department collaboration with community pharmacies in Palm Beach County, Florida for H1N1 influenza pandemic response. J. Public Health Manag. Pract..

[16] Idaho State Legislature (2011). Minutes of the House Health & Welfare Committee. https://legislature.idaho.gov/sessioninfo/2011/standingcommittees/HHEA/.

[17] Idaho State Legislature (2011). Minutes of the Senate Health & Welfare Committee. https://legislature.idaho.gov/sessioninfo/2011/standingcommittees/SHW/.

[18] Stucke J. (2008). Filling the Fluoride Gap. The Spokesman-Review. http://www.spokesman.com/stories/2008/aug/17/filling-the-fluoride-gap/.

[19] Idaho State Legislature House Bill 108. 2015 Session. https://legislature.idaho.gov/sessioninfo/2015/legislation/H0108/.

[20] Idaho State Legislature Senate Bill 1322. 2016 Session. https://legislature.idaho.gov/sessioninfo/2016/legislation/S1322/.

[21] Adams A.J. (2016). Pharmacist Prescriptive Authority for Epinephrine Auto-Injectors in Idaho. Inov. Pharm..

[22] Idaho State Legislature (2015). Minutes of the House Health & Welfare Committee. https://legislature.idaho.gov/wp-content/uploads/sessioninfo/2015/standingcommittees/0218_hhea_0900AM-Minutes.pdf.

[23] Kids with Food Allergies (2016). Idaho Mom Gets Law Passed Allowing Pharmacists to Prescribe Epinephrine. https://community.kidswithfoodallergies.org/blog/idaho-mom-gets-law-passed-allowing-pharmacists-to-prescribe-epinephrine.

[24] Idaho State Legislature Senate Bill 1294. 2016 Session. https://legislature.idaho.gov/sessioninfo/2016/legislation/S1294/.

[25] Idaho State Legislature House Bill 3. 2017 Session. https://legislature.idaho.gov/wp-content/uploads/sessioninfo/2017/legislation/H0003.pdf..

[26] Atkinson D., Dering-Anderson A.M., Adams A.J. (2017). Pharmacy-Based Tuberculosis Skin Testing (TST): Approaches to Legal Authority. Inov. Pharm..

[27] Idaho State Legislature House Bill 4. 2017 Session. https://legislature.idaho.gov/wp-content/uploads/sessioninfo/2017/legislation/H0004.pdf.

[28] Adams A.J., Suchanek Hudmon K. (2018). Pharmacist Prescriptive Authority for Tobacco Cessation Medications. J. Am. Pharm. Assoc..

[29] (2016). Idaho Health Behaviors, 2016. Results from the Behavioral Risk Factor Surveillance System. Bureau of Vital Records and Health Statistics, Division of Public Health, Idaho Department of Health and Welfare. https://www.cdc.gov/mmwr/volumes/66/ss/ss6616a1.htm.

[30] Idaho State Legislature (2017). Minutes of the Senate Health & Welfare Committee. https://legislature.idaho.gov/wp-content/uploads/sessioninfo/2017/standingcommittees/170201_sh&w_0300AM-Minutes.pdf.

[31] Idaho State Legislature (2017). House Bill 191. http://legislature.idaho.gov/wp-content/uploads/sessioninfo/2017/legislation/H0191.pdf.

[32] Taylor J.G., Joubert R. (2016). Pharmacist-led minor ailment programs: A Canadian perspective. Int. J. Gen. Med..

[33] Frost T.P., Adams A.J. (2018). Are advanced practice pharmacist designations really advanced?. Res. Soc. Adm. Pharm..

[34] Council on Credentialing in Pharmacy (2014). Credentialing and privileging of pharmacists: A resource paper from the Council on Credentialing in Pharmacy. Am. J. Health-Syst. Pharm..

[35] Firth S. (2018). AMA President: It’s Still ‘No’ to Single Payer. MedPageToday. https://www.medpagetoday.com/publichealthpolicy/healthpolicy/75775.

[36] Klepser M.E., Adams A.J., Klepser D.G. (2015). Antimicrobial stewardship in outpatient settings: Leveraging innovative physician-pharmacist collaborations to reduce antibiotic resistance. Health Secur..

[37] Akers J.M., Adams A.J., Klepser M.E. (2018). Review of Pharmacy-Based Management of Uncomplicated Urinary Tract Infections (UTIs) in Community Pharmacy Settings. Int. J. Pharm..

[38] Klepser M.E., Klepser D.G., Dering-Anderson A., Morse J.A., Smith J.K., Klepser S.A. (2016). Effectiveness of a pharmacist-physician collaborative program to manage influenza-like illness. J. Am. Pharm. Assoc..

[39] Klepser D.G., Klepser M.E., Smith J.K., Dering-Anderson A., Nelson M., Pohren L.E. (2018). Utilization of influenza and streptococcal pharyngitis point-of-care testing in the community pharmacy practice setting. Res. Soc. Adm. Pharm..

[40] Hind C. (2018). NHS Grampian project: Treating uncomplicated lower urinary tract infection in community pharmacy. Pharm. J..

[41] Beahm N.P., Smyth D.J., Tsuyuki R.T. (2018). Outcomes of Urinary Tract Infection Management by Pharmacists (RxOUTMAP): A study of pharmacist prescribing and care in patients with uncomplicated urinary tract infections in the community. Can. Pharm. J..

[42] Gauld N.J., Zeng I.S., Ikram R.B., Thomas M.G., Buetow S.A. (2017). Antibiotic treatment of women with uncomplicated cystitis before and after allowing pharmacist-supply of trimethoprim. Int. J. Clin. Pharm..

[43] Klepser M.E., Adams A.J. (2018). Pharmacy-based management of influenza: Lessons learned from research. Int. J. Pharm. Pract..

[44] MedSask Guidelines for Prescribing for Minor Ailments and Patient Self-Care. https://medsask.usask.ca/professional/guidelines/index.php.

[45] Washington State Pharmacy Association Clinical Community Pharmacist. https://www.wsparx.org/page/CCP.

[46] Chopski N. (2018). Patient Assessment Protocols. Licensees of the Idaho State Board of Pharmacy. https://bop.idaho.gov/code_rules/2018_04_13_Final%20BOP%20Protocol%20Packet.pdf.

[47] Tsuyuki R.T. (2018). FAQs (frequent asinine questions) on pharmacists’ scope of practice. Can. Pharm. J..

[48] Vanderholm T., Renner H.M., Stolpe S.F., Adams A.J. (2018). An Innovative Approach to Improving the Proposed CMS Star Rating “Statin Use in Persons with Diabetes”. J. Manag. Care Spec. Pharm..

[49] Moffett P., Moore G. (2011). The Standard of Care: Legal History and Definitions: The Bad and Good News. West J. Emerg. Med..

[50] National Association of Boards of Pharmacy (2018). Task Force to Develop Regulations Based on Standards of Care. https://nabp.pharmacy/task-force-to-develop-regulations-based-on-standards-of-care/.

[51] Adams A.J. (2018). Transitioning Pharmacy to “Standard of Care” Regulation: Analyzing how Pharmacy Regulates Relative to Medicine and Nursing. Res. Soc. Adm. Pharm..

[52] (2018). Broulim’s Pharmacies Now Offering Clinical Services in East Idaho. East Idaho News. https://www.eastidahonews.com/2018/07/broulims-pharmacies-now-offering-clinical-services-in-east-idaho/.

[53] (2018). Albertsons First Chain Pharmacy to Practice New Idaho Law. Chain Drug Review. https://www.chaindrugreview.com/albertsons-first-large-chain-pharmacy-to-practice-new-idaho-law/.

[54] Alexander A. (2018). Rite Aid’s Idaho Pharmacists Will Soon Prescribe Some Meds. DrugStoreNews. https://www.drugstorenews.com/retail-news/rite-aid-pharmacists-in-idaho-to-prescribe-select-meds-effective-july-1/.

[55] Yap D. (2018). Idaho Pharmacists Can Prescribe More than 20 Categories of Medications. Pharm. Today.

[56] Adams A.J. (2020). Rethinking Pharmacy Regulation: Core Elements of Idaho’s Transition to a “Standard of Care” Approach. J. Am. Pharm. Assoc..

